# Calcium signaling: an underlying link between cardiac disease and carcinogenesis

**DOI:** 10.1186/s13578-018-0236-0

**Published:** 2018-06-08

**Authors:** Xuehong Xu, Steven P. Balk, William B. Isaacs, Jianjie Ma

**Affiliations:** 1 Laboratory of Cell Biology, Genetics and Developmental Biology, Shaanxi Normal University College of Life Sciences, Xi’an, 710062 China; 2 Beth Israel Deaconess Medical Center, Harvard Medical School, Boston, MA 02215 USA; 3 Johns Hopkins School of Medicine, Baltimore, MD 21287 USA; 4 Ohio State University College of Medicine, Columbus, OH 43210 USA

Cardiac disease and cancer are the top two health threatening disorders, resulting in the loss of millions of lives every year. The countless spending has attempted to mitigate these losses; however, the benefits of this spending do not yet meet society satisfaction. Many research efforts are focused on elucidating the molecular mechanisms underlying carcinogenesis, including PD-1/CTLA-4, CCR, cytokines, P53, Notch and Wnt signaling pathways. Clinical trials with PD-1/CTLA-4 immune checkpoint inhibitor-based cancer therapy have been especially promising, but a major challenge is autoimmune reactions including mortality due to myocarditis [1]. Thus, more intensive study is needed to examine the impact of cancer treatment on cardiac disease.

Calcium signaling, a fundamental pathway involved in control of cardiac function and cell proliferation and malignancy, might provide a link between cardiac dysfunction and cancer progression and therapy. Calcium signaling is involved at the molecular and cellular levels in most biological processes, such as neuronal excitability, excitation–contraction coupling in the heart and skeletal muscle [2, 3], embryo fertilization and development [4], and bone formation and remodeling. Altered calcium metabolism has been linked to tumorigenesis and heart failure [5, 6].

Intracellular calcium levels are regulated by the cell membrane calcium-sensing receptor (CaSR), which detects levels of extracellular serum calcium, and inositol 1,4,5-triphosphate receptors (IP3Rs) that regulate calcium release from intracellular stores. This calcium regulation is critical for multiple functions including cell proliferation, apoptosis, fertilization, and development. In a recent study, Bononi et al. found that the tumor suppressor BAP1, previously characterized as a nuclear deubiquitinase, is also localized in the endoplasmic reticulum (ER). This ER localized BAP1 deubiquitylates and thereby stabilizes ER IP3R3, so that BAP1 loss results in decreased calcium flux and impaired apoptotic responses [5]. In another study Kuchay et al. reported that the F-box protein FBXL2 binds to and ubiquitylates IP3R3, targeting it for proteasome-mediated degradation and thereby limiting calcium influx into mitochondria and apoptosis [6]. They further found that PTEN competes with FBXL2 for IP3R3 binding, so that PTEN loss (in addition to its activation of the PI3 kinase pathway) increases the FBXL2-dependent degradation of IP3R3. In this thematic series, Wang et al. [7] and Xu et al. [8] summarize these recent findings linking the essential role of calcium signaling in normal cells to cancer progression and treatment.

Resident cardiac macrophages have multiple functions including post myocardial infarction repair, which may involve intercellular calcium signaling cross-talk between cardiomyocytes and macrophages. Hulsmans et al. discovered that resident macrophages can communicate with the atrial-ventricular node of the heart to facilitate cardiac electrical conduction [9]. Distinct gene expression patterns related to intracellular calcium signaling could contribute to the inflammatory response and contractile dysfunction of the heart. Moreover, coordinated cell programming may underlie the specific lineage of the resident macrophage in the heart. In this thematic series, Zhou et al. [10] provide a review of the literature that links calcium signaling to diverse functions in different tissues.

Resolving the spatial and temporal aspects of calcium signaling in vivo and in vitro is critical for understanding the physiology and pathology of heart disease and cancer treatment. In this thematic series, Staehlke et al. [11] designed a highly sensitive approach with an amino-group containing plasma polymer nanolayer to examine calcium ion mobilization in osteoblasts. Conjugating near-infrared indocyanine green (ICG) dyes with human serum albumin by covalently binding to gold nanorod, Zhang et al. [12] have developed a sensitive near-field fluorescence detection approach that will provide a more sensitive tool for detecting calcium signaling in vivo at the cellular level and in living animals theoretically.
